# Fast-track multidisciplinary treatment versus conventional treatment for colorectal cancer: a multicenter, open-label randomized controlled study

**DOI:** 10.1186/s12885-019-6188-x

**Published:** 2019-10-23

**Authors:** Jun Li, Xiang-Xing Kong, Jiao-Jiao Zhou, Yong-Mao Song, Xue-Feng Huang, Gen-Hai Li, Xiao-Jiang Ying, Xiao-Yu Dai, Min Lu, Kai Jiang, Dong-Liang Fu, Xin-Lin Li, Jin-Jie He, Jian-Wei Wang, Li-Feng Sun, Dong Xu, Jing-Yan Xu, Min Chen, Yu Tian, Jing-Song Li, Min Yan, Ying Yuan, Ke-Feng Ding

**Affiliations:** 1 Department of Colorectal Surgery and Cancer Institute (Key Laboratory of Cancer Prevention and Intervention, China National Ministry of Education; Key Laboratory of Molecular Biology in Medical Sciences, Zhejiang Province, China), the Second Affiliated Hospital of Zhejiang University School of Medicine, Hangzhou, China; 2 Department of Anus and Large Intestine, Sir Run Shaw Hospital, Zhejiang University College of Medicine, No. 3 East Qingchun Road, Hangzhou, 310016 Zhejiang Province China; 3 Department of Anus and Large Intestine, People’s Hospital of Yuyao, 800 City Road East, Yuyao, 315400 Zhejiang Province China; 4 Department of Anorectum, People’s Hospital of Shaoxing, 568 Zhong-Xing North Rd, Shaoxing, 312000 Zhejiang Province China; 5 Department of Anus and Large Intestine, Ningbo No. 2 Hospital, No. 41 Northwest Road, Ningbo, 315010 Zhejiang Province China; 6 Department of Anus and Large Intestine, Second Affiliated Hospital, Wenzhou Medicine College, 109 Xue-Yuan West Rd, Wenzhou, 325027 Zhejiang Province China; 7 Department of Anesthesiology, Second Affiliated Hospital, Zhejiang University School of Medicine, No. 88 Jiefang Road, Hangzhou, 310009 Zhejiang Province China; 8 Engineering Research Center of EMR and Intelligent Expert System, Ministry of Education, Collaborative Innovation Center for Diagnosis and Treatment of Infectious Diseases, College of Biomedical Engineering and Instrument Science, Zhejiang University, No. 38 Zheda Road, Hangzhou, 310027 Zhejiang China; 9 Department of Medical Oncology, Second Affiliated Hospital, and The Key Laboratory of Cancer Prevention and Intervention, China National Ministry of Education, Zhejiang University School of Medicine, No. 88 Jiefang Road, Hangzhou, 310009 Zhejiang Province China

**Keywords:** Colorectal surgery, Rehabilitation, Colorectal cancer, Randomized controlled trial

## Abstract

**Background:**

Laparoscopic surgery, fast-track perioperative treatment and XELOX chemotherapy are effective strategies for shortening the duration of hospital stay for cancer patients. This trial aimed to clarify the safety and efficacy of the fast-track multidisciplinary treatment (FTMDT) model compared to conventional surgery combined with chemotherapy in Chinese colorectal cancer patients.

**Methods:**

This trial was a prospective randomized controlled study with a 2 × 2 balanced factorial design and was conducted at six hospitals. Patients in group 1 (FTMDT) received fast-track perioperative treatment and XELOX adjuvant chemotherapy. Patients in group 2 (conventional treatment) received conventional perioperative treatment and mFOLFOX6 adjuvant chemotherapy. Subgroups 1a and 2a had laparoscopic surgery and subgroups 1b and 2b had open surgery. The primary endpoint was total length of hospital stay during treatment.

**Results:**

A total of 374 patients were randomly assigned to the four subgroups, and 342 patients were finally analyzed, including 87 patients in subgroup 1a, 85 in subgroup 1b, 86 in subgroup 2a, and 84 in subgroup 2b. The total hospital stay of group 1 was shorter than that of group 2 [13 days, (IQR, 11–17 days) vs. 23.5 days (IQR, 15–42 days), *P* = 0.0001]. Compared to group 2, group 1 had lower surgical costs, fewer in-hospital complications and faster recovery (all *P* < 0.05). Subgroup 1a showed faster surgical recovery than that of subgroup 1b (all *P* < 0.05). There was no difference in 5-year overall survival between groups 1 and 2 [87.1% (95% CI, 80.7–91.5%) vs. 87.1% (95% CI, 80.8–91.4%), *P* = 0.7420].

**Conclusions:**

The FTMDT model, which integrates laparoscopic surgery, fast-track treatment, and XELOX chemotherapy, was the superior model for enhancing the recovery of Chinese patients with colorectal cancer.

**Trial registration:**

ClinicalTrials.gov: NCT01080547, registered on March 4, 2010.

## Background

Globally, colorectal cancer is the third most common malignancy [1, 2]. In 2015, there were nearly 376,000 Chinese patients diagnosed with colorectal cancer [3]. Most of these patients could have been cured by radical surgery with or without perioperative chemotherapy and radiotherapy. Fast-track surgery is a combination of several evidence-based perioperative interventions to enhance the recovery of patients after surgery [4]. Studies on fast-track surgery have shown that many conventional perioperative procedures (e.g., bowel preparation and long preoperative fasting) are unnecessary or even harmful to colorectal cancer patients [5, 6].

Two European clinical trials, “EnROL” and “LAFA”, have shown that fast-track surgery is safe and effective for colorectal cancer patients [7, 8]. In both trials, fast-track laparoscopic surgery was the best choice in terms of postoperative hospital stay. Furthermore, four Chinese prospective studies have reported that fast-track surgery effectively accelerates early recovery and reduces the postoperative hospital stay for colorectal cancer patients [9–12]. However, three of the trials have small study populations. Moreover, none of the four studies reported the perioperative procedural details. At present, all reported fast-track surgery studies for colorectal cancer have detailed only the postoperative period (usually only 1 week). However, two-thirds of the patients required 6 months of postoperative adjuvant chemotherapy. Additionally, some procedures in the LAFA and EnROL trials were considered by Chinese surgeons to be radical and were difficult to comply with.

Therefore, we proposed the fast-track multidisciplinary treatment (FTMDT) model in 2010 [13]. This model modifies the fast-track surgical protocols, which are conservative and easy for Chinese surgeons and patients to comply with. Moreover, FTMDT includes fast-track surgery and subsequent adjuvant chemotherapy with capecitabine and oxaliplatin (XELOX). FTMDT can enhance the whole rehabilitation process for patients with colorectal cancer compared to conventional treatment consisting of conventional surgery followed by adjuvant chemotherapy with leucovorin, fluorouracil, and oxaliplatin (FOLFOX). The FTMDT model, which includes more conservative surgical procedures than those in Western countries and covers the overall treatment process, is novel and has never been prospectively compared with conventional treatment. Therefore, this randomized trial aims to compare the safety and efficacy of the FTMDT model versus the conventional model for Chinese patients with colorectal cancer. Moreover, this trial aimed to investigate the total length of hospital stay for patients who received laparoscopic fast-track surgery compared to those who underwent open fast-track surgery.

## Methods

### Patients, study design, and randomization

This was an open-label, prospective randomized controlled study with a 2 × 2 balanced factorial design (Clinicaltrials.gov NCT01080547). Eligible patients were randomized (1:1:1:1) to receive either laparoscopic fast-track surgery followed by XELOX (group 1a), open fast-track surgery followed by XELOX (group 1b), laparoscopic conventional surgery followed by FOLFOX (group 2a), or open conventional surgery followed by FOLFOX (group 2b). Prof. JS L and Dr. Y T, College of Biomedical Engineering and Instrument Science Zhejiang University, oversaw distribution of patients into four study subgroups (1:1:1:1) by simple randomization according with the random number table without stratification. Each participating study center screened and recruited patients. The baseline information was reported to Prof. JS L and Dr. Y T. They performed the patient randomization and informed every center of the randomization results. This trial was approved by the Ethics Committee of Second Affiliated Hospital Zhejiang University School of Medicine (2010LSY No. 6).

The inclusion criteria were patients ≥18 years old with pathologically confirmed colon or upper rectal (distance between the tumor lower margin and anus > 12 cm) cancer. All patients were also screened by the investigators and signed informed consents. The exclusion criteria were patients with tumors that could be removed by endoscopic mucosal resection or patients who had a history of malignancy, bowel obstruction, intestinal perforation, evidence of metastasis through physical examination and/or radiological examination, acute disease, acute attack of chronic disease, psychiatric history, spinal deformity that was contraindicated for epidural anesthesia, an American Society of Anesthesiologists (ASA) score IV or higher, or mid-low rectal cancer, or patients who were pregnant.

### Study endpoints

The primary endpoint was the total duration of hospital stay from the time of randomization to 30 days after the last cycle of postoperative chemotherapy. Therefore, it included the days of hospital stay for surgery, adjuvant chemotherapy, and readmission. The postoperative discharge criteria were (1) good pain control (numeric rating scale ≤3), (2) tolerance of solid food, and (3) recovery of independent activities of daily living to the patient’s preoperative level.

Secondary endpoints included (1) quality of life assessed before surgery and at 1 week, 3 months, and 6 months after surgery via European Organization for Research and Treatment (EORTC) QLQ-C30 and QLQ-CR38 questionnaires; (2) the number of patients with chemotherapy-related adverse events according to the National Cancer Institute Common Terminology Criteria for Adverse Events (NCI CTCAE Version 3.0), which was measured up to 30 days after the last administration of chemotherapy; (3) the number of patients with intraoperative and postoperative (measured up to 30 days postoperative) surgical complications, e.g., infection of the incision site, anastomotic leakage, and readmission; and (4) the medical costs (RMB), associated with the whole hospitalization measured up to 30 days after the last surgical procedure or chemotherapy treatment.

Some secondary endpoints that were not prespecified in the study protocol were also analyzed. The surgery duration was calculated as the time from the initial skin incision to the closing of the abdomen. Blood loos was calculated as the blood lost from the time of initial skin incision to the closing of the abdomen. Ambulation onset was recorded as the first time that patient got out of bed postoperatively. Some additional recovery characteristics included the times to first flatus, to defecation, and to resume fluid diet and the duration of the postoperative hospital stay. Thirteen perioperative characteristics, including psychological optimism, anesthesia information, laparoscopy-guided examination, bowel preparation, fasting and oral intake, epidural anesthesia, warming, abdominal drains, fluid infusion, diet, intravenous fluid infusion, nasogastric tube, urethral catheter, and ambulation were assessed to evaluate treatment compliance. Patients who violated more than 10 checkpoints were considered to have not received the allocated intervention. Disease-free survival (DFS) was calculated as the time from randomization to recurrence or death. Overall survival (OS) was the time to death for any reason. All of the above endpoints were compared between groups 1 and 2 to clarify the superiority of FTMDT model over the conventional treatment model. Additionally, all of the above endpoints were compared between subgroups 1a and 1b to clarify the superiority of laparoscopy over open surgery within the set of fast-track surgery procedures.

The FTMDT trial initially included three participating centers, the including Second Affiliated Hospital Zhejiang University School of Medicine, People’s Hospital of Shaoxing, and the Second Affiliated Hospital Wenzhou Medicine College. Three additional centers joined in this trial in 2012 to enhance patient recruitment. The new centers were Sir Run Shaw Hospital of Zhejiang University School of Medicine, Ningbo No. 2 Hospital, and People’s Hospital of Yuyao. All surgeons taking part in this trial had performed more than 20 laparoscopic operations for colorectal cancer as suggested by the American Society of Colon and Rectal Surgeons [14]. Paper case report forms (CRFs) were collected by the investigators of every participating centers. The CRFs were then collected by the primary investigator Prof. Ding when the patients finished the whole treatment. The investigators of each participating center took responsibility for updating the follow-up data.

### Procedures

The interventions for each group have been previously described in detail [13]. Briefly, patients in group 1 (FTMDT) were given enhanced recovery procedures and 8 cycles of XELOX for high-risk stage II or stage III colorectal cancer. Patients in group 2 (conventional treatment) were given conventional perioperative care and 12 cycles mFOLFOX6 for high-risk stage II or stage III colorectal cancer. The hospital stay for postoperative chemotherapy was 1 day for XELOX and 3 days for mFOLFOX6.

### Sample size

We estimated that the overall duration of the hospital stay for subgroups 1a and 1b would be 14 and 16 days, respectively. Base on our previous research, the overall duration of the hospital stay of groups 2a and 2b were predicted as 46 and 48 days, respectively [15]. With a standard deviation of 6 days for the mean number of hospitalization days, a total sample size of 218 patients would have a power of > 0.85 to detect a minimum reduction in hospital stay of 2 days among the four groups, using a 5% significance level. The patients with high-risk stage II or stage III disease who needed adjuvant chemotherapy accounted for 64% of the total colorectal cancer patients [16]. Therefore, a total of 340 patients, with 85 in each group, were necessary. Considering a 10% drop-out rate, we planned to recruit 372 patients for randomization to the four subgroups.

### Statistical analysis

Data were analyzed according to the principle of intention to treat. Normal continuous data were presented as the means ± standard deviations and compared between groups by analysis of variance (ANOVA, > 2 groups) or unpaired t-test (2 groups). Non-normal distribution data are presented as the medians and interquartile ranges (IQR) and were compared between groups by the Mann-Whitney U test or the Kruskal-Wallis test. Categorical data were compared between groups by the χ2 test or Fisher’s exact test for probability. The reported follow-up results were based on the data collected through February 27, 2019. Kaplan-Meier curves of OS and DFS were compared between groups by using the log-rank test. A two-sided *P*-value of 0.05 or less indicated statistical significance. A median difference of more than 10 points in quality of life scoring represented a clinically significant difference [17]. All analyses were performed using STATA (version 12.0; STATA, College Station, TX, USA).

## Results

### Patient population

**Fig. 1:**
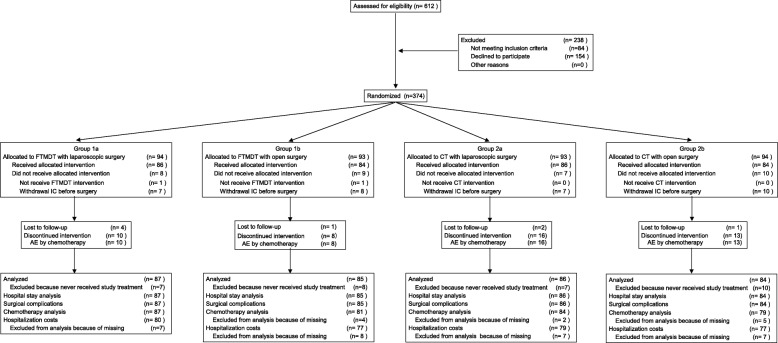
CONSORT flow diagram. *FTMDT* fast-track multidisciplinary treatment, *CT* conventional treatment, *IC*, informed consent

From April 2010 through June 2014, 612 patients were screened. A total of 374 patients were randomly assigned to the four subgroups. Thirty-two patients refused the assigned intervention and withdrew informed consent before surgery. A total of 342 patients were finally analyzed, including 87 patients in subgroup 1a, 85 in subgroup 1b, 86 in subgroup 2a, and 84 in subgroup 2b (Fig. 1). The baseline patients’ demographic and clinical characteristics were balanced between groups 1 and 2. The maximum tumor diameter in subgroup 1a was lower than that in subgroup 1b (*P* = 0.0084). The distribution of pT stages was unequal between subgroup 1a and 1b (*P* = 0.0210). During surgery, a patient in subgroup 1a was found to have peritoneal metastasis (Table 1).

**Table 1:** Baseline Patient Demographic and Clinical Characteristics

Characteristic	Groups		*P* value	Group1 (FTMDT)		*P* value
	1(FTMDT)	2(Conventional)		Subgroup 1a	Subgroup 1b	
Age, years (M, IQR)	60 (53–66.5)	61 (54–69)	0.3415	61 (52–66)	60 (54–67)	0.7559
Gender, F/M (F%)	62/110 (36)	67/103 (39)	0.5210	30/57 (34)	32/53 (38)	0.6660
BMI, M (IQR)	22.6 (20.8–24.6)	22.67 (20.6–24.84)	0.9457	22.1 (21.2–24.8)	23.02 (20.65–24.55)	0.7215
ASA grade, n (%)			0.3920			0.6340
I	156 (91)	148 (87)		78 (90)	78 (92)	
II	16 (19)	21 (12)		9 (10)	7 (8)	
III	0 (0)	1 (1)		0 (0)	0 (0)	
CEA, ng/mL, M (IQR)	6.7 (3.8–15.2)	5.6 (2.9–12.1)	0.3007	6.1 (3.1–12.9)	7.0 (4.8–20.8)	0.2561
CA199, U/mL, M, (IQR)	28.1 (7.0–77.8)	29.8 (7.0–51.0)	0.6492	16.8 (6.1–57.0)	28.3 (10.5–103.2)	0.1482
Site of Cancer, n (%)			0.5110			0.8810
Ascending colon	31 (18)	35 (21)		18 (21)	13 (15)	
Transverse colon	13 (8)	12 (7)		6 (7)	7 (8)	
Descending colon	20 (12)	24 (14)		11 (13)	13 (15)	
Sigmoid colon	50 (29)	38 (22)		24 (28)	26 (31)	
Rectum	54 (33)	65 (36)		28 (32)	26 (31)	
Maximum tumor diameter, cm, M (IQR)	4 (3.5–5)	4 (3–5)	0.3358	4 (3–5)	5 (4–6)	0.0084
Pathologic type, n (%)						0.1400
Adenocarcinoma	148 (86)	146 (86)	0.6260	73 (84)	75 (88)	
Mucinous adenocarcinoma	9 (5)	13 (8)		6 (6.90)	3 (4)	
Other	3 (2)	2 (1)		3 (3)	0 (0)	
Missing	12 (7)	9 (5)		5 (6)	7(8)	
Differentiation, n (%)			0.3840			0.8480
Well	20 (12)	20 (12)		11 (13)	9 (11)	
Moderate	104 (60)	115 (68)		51 (59)	53 (62)	
Poor	34 (20)	25 (15)		18 (21)	16 (19)	
Missing	14 (8)	10 (5)		7 (8)	7 (8)	
Lymph nodes, M (IQR)	15 (11–20)	15 (12–18)	0.7319	14 (9–19)	16 (11–21)	0.3270
pT stage, n (%)			0.8070			0.0210
1	9 (5)	9 (5)		7 (8)	2 (2)	
2	27 (16)	22 (13)		11 (13)	16 (19)	
3	91 (53)	96 (56)		53 (61)	38 (45)	
4	33 (19)	28 (16)		11 (13)	22 (26)	
Missing	12 (7)	15 (10)		5 (6)	7 (8)	
pN stage, n (%)			0.5360			0.5620
0	97 (56)	88 (52)		48 (55)	49 (58)	
1	39 (23)	46 (27)		23 (26)	16 (19)	
2	25 (15)	21 (12)		12 (14)	13 (15)	
Missing	11 (6)	15 (9)		4 (5)	7 (8)	
pTNM stage (n, %)			0.7110			0.6720
I	27 (16)	23 (14)		13 (15)	14 (16)	
II	66 (38)	63 (37)		32 (37)	34 (40)	
III	66 (38)	69 (41)		36 (41)	30 (35)	
IV	1 (1)	0 (0)		1 (1)	0 (0)	
Missing	12 (7)	15 (8)		5 (6)	7 (9)	

*FTMDT* Fast-track multi-discipline treatment, *M (IQR)* Median (interquartile range), *F/M* Female/male, *F%* Percent of females in subgroup, *BMI* Body mass index, *ASA* American Society of Anesthesiologists, *n* number, *CEA* Carcinoembryonic antigen, *CA199* Carbohydrate antigen 19–9, *pT* pathological T stage, *pN* pathological N stage, *pTNM* pathological 7th edition TNM stage

### Hospital stay, compliance, surgical recovery, chemotherapy and costs

The primary endpoint of total hospital stay was shorter in group 1 than in group 2 (13 days vs. 23.5 days, *P* = 0.0001). The total hospital stay of subgroup 1a was similar to that of subgroup 1b (13 days vs. 14 days, *P* = 0.1951, Table 2).

**Table 2:** Primary endpoint and secondary endpoints

Characteristic	Groups		*P* value	Group1 (FTMDT)		*P* value
	1(FTMDT)	2(Conventional)		Subgroup 1a	Subgroup 1b	
Total in-hospital days, M (IQR)	13 (11–17)	23.5 (15–42)	0.0001	13 (11–16)	14 (12–17)	0.1951
Surgery						
Surgery duration, min, M (IQR)	155 (129–180)	150 (122–183)	0.8005	155 (140–180)	150 (120–180)	0.1028
Blood loss, mL, M (IQR))	100 (50–200)	150 (100–200)	0.0014	100 (50–150)	100 (100–200)	0.0150
Intraoperative complications, n (%)	2 (1.2)	1 (0.6)	1.0000	0 (0.0)	2 (2.4)	0.1480
Postoperative in-hospital complications, n (%)	11 (6.4)	25 (14.7)	0.0140	5 (5.7)	6 (7.1)	0.7700
Wound infection	1 (1)	0 (0)	1.0000	1 (1)	0 (0)	1.0000
Anastomotic leakage	0 (0)	1 (1)	1.0000	0 (0)	1 (1)	1.0000
Readmission < 30 days, n (%)	10 (5.8)	9 (5.3)	0.8340	6 (6.9)	4 (4.7)	0.5390
Ambulation onset, h, M, (IQR)	24 (24–48)	48 (48–72)	0.0001	24 (24–48)	48 (24–48)	0.1064
Days to flatus, M *± SD*	56 *± 26*	71 *± 27*	*< 0.0001*	51 *± 25*	62 *± 26*	0.0121
Days to defecation, M (IQR)	85 (55–97)	96 (69–129)	0.0010	76 (48–96)	90 (72–103)	0.0345
Days to fluid diet resumption, M (IQR)	24 (24–48)	96 (72–120)	0.0001	24 (24–24)	24 (24–48)	0.1624
Postoperative days, M (IQR)	6 (5–7)	9 (7–11)	0.0001	6 (4–8)	6 (5–7)	0.2160
Surgical protocol compliance, M (IQR)	9 (8–10)	12 (12–12)	0.0001	9 (8–10)	9 (8–10)	0.2235
Surgical costs, RMB, M (IQR)	29,678 (25868–35,045)	33,559 (29627–41,452)	0.0001	32,811(28062–37,117)	27,156(24490–32,684)	0.0001
Chemotherapy						
Patients received chemotherapy, n (%)	105 (62.5)	103 (63.2)	0.9100	55 (63.2)	50 (61.7)	0.8740
Patients with any grade AEs, n (%)	94 (94.0)	92 (96.8)	0.4990	48 (92.3)	46 (95.8)	0.6790
Patients with grade 3–4 AEs, n (%)	28 (28.0)	28 (29.5)	0.875	14 (26.9)	14 (29.2)	0.8270
Cost for chemotherapy, RMB, M (IQR)	100,999 (59021–115,102)	104,256 (59954–128,233)	0.1410	100,414 (38221–117,460)	102,353 (64528–113,722)	0.7274
Survival						
5-year DFS (%, 95CI)	82.6 (75.6–87.8)	80.0 (73.0–85.4)	0.2780	81.2 (70.8–88.3)	83.7 (72.8–90.5)	0.7560
5-year OS (%, 95CI)	87.1 (80.7–91.5)	87.1 (80.8–91.4)	0.7420	83.7 (73.6–90.2)	90.7% (81.5–95.5)	0.2540

*FTMDT* Fast-track multi-discipline treatment, *M (IQR)* Median (interquartile range), *M* ± *SD* Mean ± standard deviation, *n* number, *h* hour, *AEs* Adverse events, *RMB* Ren Min Bi, *DFS* Disease free survival, *OS* Overall survival, *CI* Confidence interval

The median number of surgical checkpoints for which the actual procedures carried out were compliant with the planned procedures was lower in group 1 than in group 2 (9 vs. 12, out of 13 checkpoints, *P* = 0.0001). The postoperative hospital stay was shorter for group 1 than for group 2 (6 days vs. 9 days, *P* = 0.0001). There was no difference between subgroups 1a and 1b in postoperative hospital stay (6 days vs. 6 days, *P* = 0.2160). The open operation performed with the fast-track protocol (subgroup 1b) resulted in shorter postoperative hospital stays than did the laparoscopic operation performed with the conventional treatment (subgroup 2a) (6 days vs. 8 days, *P* = 0.0001). The times to resumption of flatus and first defecation were earlier in group 1 than in group 2 (*P* < 0.05). The times for subgroup 1a were earlier than those in subgroup 1b (*P* < 0.05). The times to resumption of a fluid diet and to ambulation were shorter in group 1 than in group 2 (*P* < 0.01, Table 2).

The morbidity of intraoperative complications was similar between groups 1 and 2 (*P* = 1.0000). The volume of blood loss was lower in group 1 than in group 2 (100 ml vs. 150 ml, *P* = 0.0014). The volume of blood loss in subgroup 1a was lower than that in subgroup 1b (*P* = 0.0150). The morbidity of postoperative complications was lower in group 1 than in group 2 (6.4% vs. 14.7%, *P* = 0.0140), and there was no significant difference between subgroups 1a and 1b. The readmission rates during the 30 days after surgery were similar for groups 1 and 2 (5.8% vs. 5.3%, *P* = 0,8340). The surgical cost in group 1 was lower than that in group 2 (29,678 RMB vs. 33,559 RMB, *P* = 0.0001). The surgical cost for subgroup 1a was greater than that for subgroup 1b (*P* = 0.0001, Table 2). The open fast-tract surgery (subgroup 1b) generated the lowest surgical costs among all four subgroups in the FTMDT trial.

The percent of patients who received adjuvant chemotherapy was similar between groups 1 and 2 (62.5% vs. 63.2%, *P* = 0.9100). The morbidity of all grades of adverse events was similar between the two groups (94.0% vs. 96.8%, *P* = 0.4990). One patient in subgroup 1a and one in subgroup 1b died due to cancer metastasis during adjuvant chemotherapy. The chemotherapy cost was similar between groups 1 and 2 (100,999 RMB vs. 104,256 RMB, *P* = 0.1410, Table 2).

### Quality of life

The preoperative response rate of questionnaires was higher in group 1 than in group 2 (83.7% vs. 72.4%, *P* = 0.0130). The postoperative questionnaire response rates between the two groups were similar at 1 week, 3 months, and 6 months (all *P* > 0.05) The QLQ-C30 physical functioning scores 1 week after surgery were better in group 1 than in group 2 (80 vs. 66.67, *P* = 0.0472). The QLQ-C30 fatigue scores 1 week after surgery were also better in group 1 than in group 2 (33.33 vs. 44.44, *P* = 0.0095).

### Survival

**Fig. 2:**
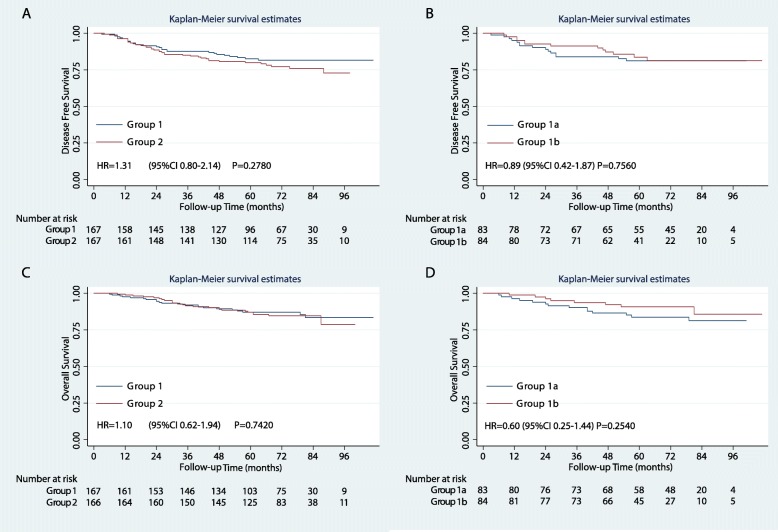
**a** Disease-free survival of the two treatment groups, **b** disease-free survival of the four subgroups, **c** overall survival of the two treatment groups, and **d** overall survival of the four subgroups in the intention-to-treat population. HR, hazard ratio; CI, confidence interval

The median follow-up time was 71 months, with no differences in DFS or OS between the treatment groups (Fig. 2, Table 2). The five-year DFS for groups 1 and 2 were 82.6% [95% confidence interval (CI), 75.6–87.8%] and 80.0% (95%CI, 73.0–85.4%), respectively (*P* = 0.2780). The five-year OS rates of groups 1 and 2 were 87.1% (95%CI, 80.7–91.5%) and 87.1% (95%CI 80.8–91.4%), respectively (*P* = 0.7420) (see in Fig. 2).

## Discussion

The concept of FTMDT was the first to integrate medical oncology with a multidiscipline treatment model [13]. This approach means that colorectal cancer is treated as an integrated disease to be followed through a recovery period of 6 months instead of just as a surgical disease. Regarding the primary endpoint, the FTMDT model decreased the total hospital stay. Moreover, the postoperative hospital stay was also shorter in the FTMDT model than in the conventional model. The results are consistent with those of previous studies.^8,10–13^ Within the scope of fast-track surgery procedures, laparoscopic surgery did not reduce the postoperative hospital stay compared to open surgery. The FTMDT trial found that laparoscopic surgery resulted in faster surgical recovery than open surgery did. The morbidity of postoperative in-hospital complications was lower in the FTMDT group than in the conventional treatment group. The five-year DFS and OS were similar between the two groups, which means that the FTMDT model for colorectal cancer is as safe as the conventional treatment model is.

The fast-track surgery protocols used in the FTMDT trial, which were significantly different from the protocols of trials conducted in Western countries, were modified for Chinese patients [13, 18, 19]. Though the concept of fast-track surgery was proposed nearly two decades ago,^4,26^ the practice of the Western model in China was difficult because of the intense doctor-patient relationship and the deep-rooted health-preserving culture in China [5, 6, 20]. Some procedures used in the LAFA and EnROL trials were considered by Chinese surgeons to be radical and possibly dangerous. These included actions on the first day after surgery, such as the oral intake of more than 2 l of liquid, intake of a normal diet, stoppage of intravenous infusion, and getting out bed for more than 6 h [18, 19]. Consequently, mandatory changes in the fast-track surgery model were tailored for Chinese patients and surgeons. The current trial verified the safety of the FTMDT model for Chinese patients. The FTMDT model, using modified and moderate fast-track surgery procedures, reduced the postoperative hospital stay just as the fast-track models in Western trials did. Our results show that fast-track surgery procedures are adaptable to various societies and cultures.

The role of laparoscopic surgery in fast-track protocols is controversial. In contrast, with the LAFA and EnROL trials, we did not find that the laparoscopic operation with FTMDT decreased the postoperative hospital stay compared to the open operation. Additionally, the postoperative hospital stay of 6 days was slightly longer than that in the LAFA/EnROL trials, which was 5 days. This result could be due to differences in the fast-track treatment procedures. Importantly, the open operation performed with the fast-track protocol resulted in shorter postoperative and overall hospital stays than did the laparoscopic operation performed with the conventional treatment. The ongoing trial known as “TAPAS”, a prospective cohort study for patients with colon carcinomas, seeks to determine which of three protocols, i.e., traditional open surgery, open fast-track surgery, and laparoscopic fast-track surgery with multimodal management, best minimizes the cost [21]. The open fast-tract surgery generated the lowest surgical costs among all four subgroups in the FTMDT trial. Similar results have also been reported by the LAFA trial.^8^ In that trial, open surgery and fast-track procedures achieved a median postoperative hospital stay of 6 days, similar to that achieved with laparoscopy and standard care. In addition, the number of days to attain preoperative levels of solid food tolerance, passage of first flatus, and mobility following open surgery with fast-track procedures was shorter than that with laparoscopy and standard care [8]. Thus, an open operation combined with fast-track treatment is a better choice than laparoscopic operation alone is.

Even though laparoscopic surgery did not significantly reduce the hospital stay more than that required for open surgery in this trial, at least three advantages still back laparoscopy as the best choice for fast-track surgery. First, compared to the open operation, the laparoscopic operation optimized by fast-tract surgical protocols resulted in much less trauma, e.g., less blood loss and reduced time to resumption of flatus and defecation. As a result, laparoscopic surgery decreased the surgical stress and accelerated postoperative nutrition and resumption of immune levels compared to open surgery [22, 23]. Second, laparoscopy ensured that surgeons could proficiently dissect tumors with a high-definition view, thus minimizing the possibility of inadvertent injury. The last but not least advantage of laparoscopy surgery is that it was welcomed by patients. In the EnROL trial, 32% of potential patients rejected recruitment because they wanted to receive laparoscopy instead of being randomized to the open surgery group [22]. This same concern by patients also slowed recruitment for the FTMDT trial in the first 2 years.

There were several limitations in this trial. First, the fast-track surgery procedures were more conservative than the Western procedures are. The median number of checkpoints that met compliance for fast-track treatment in this trial was 9 of 13 surgical checkpoints; thus only 69.2% of the fast-track procedures were complied with by the patients and surgeons. Second, the FTMDT perioperative treatment was affected by new and better understandings of perioperative procedures that were acquired during the trial itself. For instance, both groups of patients should have received bowel preparation as required by the protocol; however, only 64.3% patients in the FTMDT group received it. Third, the recruited patients in this trial were younger than the patients in the Western trials. The median age of colorectal cancer onset in China is approximately 10 years earlier than it is in Western countries [3]. The ASA scores, BMI, and morbidity of postoperative complications were lower in our trial than in the Western trials. Fourth, this trial was not conducted using a blinded protocol which may have contributed to intervention bias. For statistics, only simple randomization was adopted without stratification. The study involved both surgery and adjuvant chemotherapy, with four subgroups making the design complex and potentially imbalanced. Considering the type I error wasn’t adjusted by 2 groups, the sample size may not be enough to explain the secondary end points as there were also many confounding factors.

While the fast-track treatment with open surgery had some economic advantages, the laparoscopic surgery had minor advantages over open surgery for postoperative recovery. The integration of laparoscopic surgery, fast-track treatment, and XELOX chemotherapy in FTMDT represents an optimal model to enhance patient recovery from surgical resection of colorectal cancer.

## Conclusions

The FTMDT model, which integrates laparoscopic surgery, fast-track treatment, and XELOX chemotherapy, was the superior model for enhancing the recovery of Chinese patients with colorectal cancer.

## Data Availability

The present article is a RCT research, and the data contained identifying/confidential patient data so it is no available.

## Abbreviations

ASA: American Society of Anesthesiologists
BMI: Body mass index
CI: Confidence interval
CRFs: Case report forms
DFS: Disease-free survival
EORTC: European Organization for Research and Treatment
FOLFOX: Adjuvant chemotherapy with leucovorin, fluorouracil, and oxaliplatin
FTMDT: Fast-track multidisciplinary treatment
IQR: Interquartile ranges
NCI CTCAE: National Cancer Institute Common Terminology Criteria for Adverse Events
OS: Overall survival
XELOX: Adjuvant chemotherapy with capecitabine and oxaliplatin
